# Complete genome sequence of *Anabaena variabilis* ATCC 29413

**DOI:** 10.4056/sigs.3899418

**Published:** 2014-01-01

**Authors:** Teresa Thiel, Brenda S. Pratte, Jinshun Zhong, Lynne Goodwin, Alex Copeland, Susan Lucas, Cliff Han, Sam Pitluck, Miriam L. Land, Nikos C Kyrpides, Tanja Woyke

**Affiliations:** 1 Department of Biology, University of Missouri-St. Louis, St. Louis, MO; 2 DOE Joint Genome Institute, Walnut Creek, CA; 3 Lawrence Livermore National Laboratory, Livermore, CA; 4 Lawrence Berkeley National Laboratory, Berkeley, CA; 5 Los Alamos National Laboratory, Los Alamos, NM; 6 Oak Ridge National Laboratory, Oak Ridge, TN

## Abstract

*Anabaena variabilis* ATCC 29413 is a filamentous, heterocyst-forming cyanobacterium that has served as a model organism, with an extensive literature extending over 40 years. The strain has three distinct nitrogenases that function under different environmental conditions and is capable of photoautotrophic growth in the light and true heterotrophic growth in the dark using fructose as both carbon and energy source. While this strain was first isolated in 1964 in Mississippi and named *Anabaena flos-aquae* MSU A-37, it clusters phylogenetically with cyanobacteria of the genus *Nostoc*. The strain is a moderate thermophile, growing well at approximately 40^°^ C. Here we provide some additional characteristics of the strain, and an analysis of the complete genome sequence.

## Introduction

*Anabaena variabilis* ATCC 29413 (=IUCC 1444 = PCC 7937) is a semi-thermophilic, filamentous, heterocyst-forming cyanobacterium. Heterocysts, which are specialized cells that form in a semi-regular pattern in the filament, are the sites of nitrogen fixation in cells grown in an oxic environment. *A. variabilis* ATCC 29413 was first isolated as a freshwater strain in 1964 in Mississippi by R.G. Tischer, who called the strain *Anabaena flos-aquae* A-37 [1]. He was primarily interested in the extracellular polysaccharide produced by this strain [2-4], which was subsequently called *Anabaena variabilis* by Healey in 1973 [5]. It was characterized in more detail by several labs in the 1960’s and 1970’s [6-8]. In particular, the early work by Wolk on this strain led to its becoming a model strain for cyanobacterial physiology, nitrogen fixation and heterocyst formation [9-13]. Here we present a summary classification and a set of features for *A. variabilis* ATCC 29413 together with the description of the complete genomic sequencing and annotation.

## Classification and features

**Figure 1:**
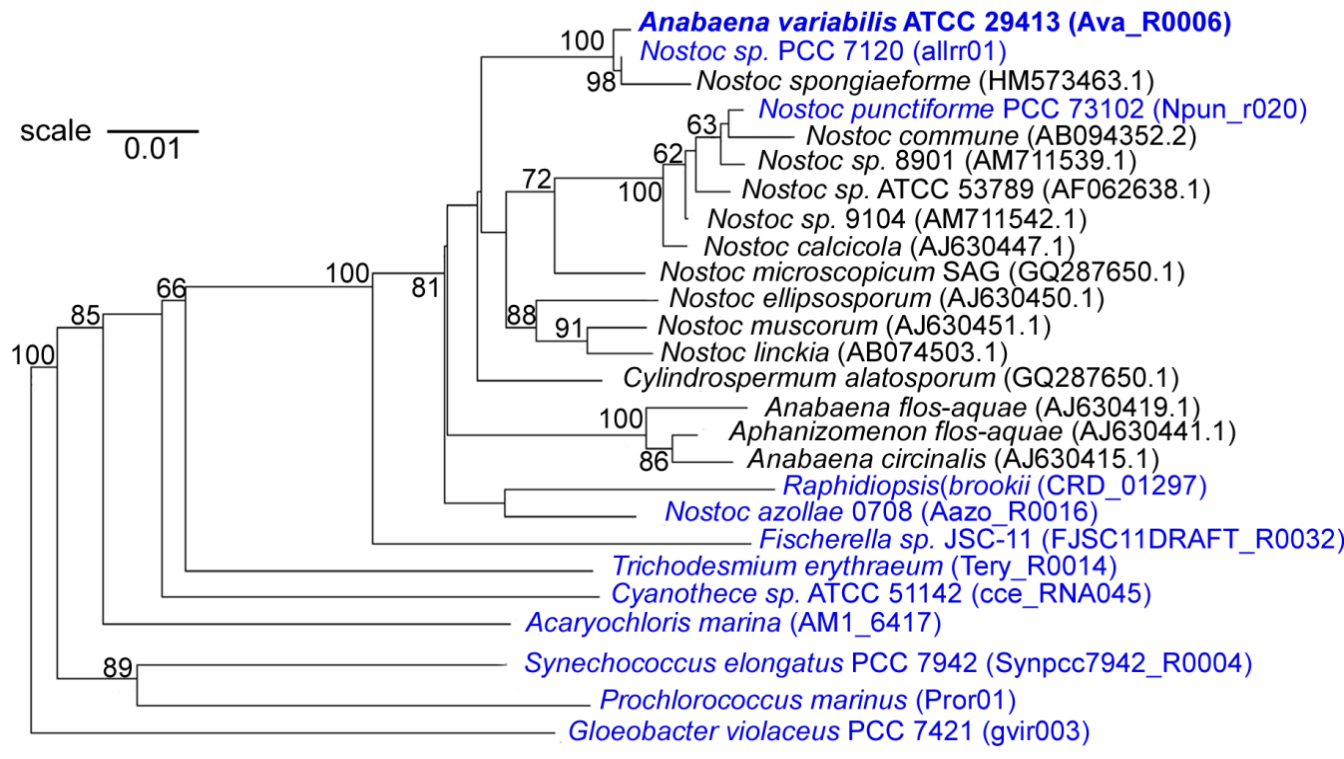
16S phylogenetic tree highlighting the position of *Anabaena variabilis* ATCC 29413 relative to other cyanobacterial strains. Strains in blue have been sequenced and the 16S rRNA IMG locus tag is shown in parentheses after the strain. GenBank accession numbers are provided for 16S rRNA genes in strains that do not have a complete genome sequence (shown in black). The tree was made with sequences aligned by the RDP aligner, with the Jukes-Cantor corrected distance model, to construct a distance matrix based on alignment model positions, without alignment inserts, and uses a minimum comparable position of 200. The tree is built with RDP Tree Builder, which uses Weighbor [26] with an alphabet size of 4 and length size of 1,000. Bootstrapping (100 times) was used to generate a majority consensus tree [27]. Bootstrap values over 60 are shown. *Gleothece violaceus* PCC 7421 was used as the outgroup.

**Figure 2:**
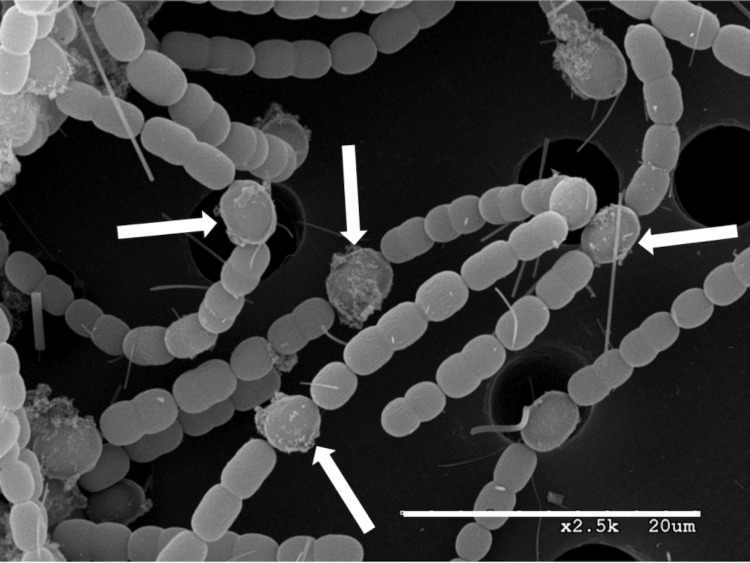
Scanning electron micrograph of filaments of *A. variabilis*. Heterocysts are the larger cells with extracellular polysaccharide visible (indicated by the white arrows). Slender fibers are an artifact caused by the use of a glass fiber filter to support the cells on the membrane filter during washing. The length of the line is 20 μm.

The general characteristics of *A. variabilis* are summarized in Table 1 and its phylogeny is shown in Figure 1. Vegetative cells of *A. variabilis* are oblong, 3-5 µm in length, have a Gram-negative cell wall structure, are normally non-motile, and form long filaments. Under conditions of nitrogen deprivation, certain vegetative cells differentiate heterocysts, which are the sites of aerobic nitrogen fixation (reviewed in [19,28,29]). Heterocysts, which comprise 5-10% of the cells in a filament, are terminally differentiated cells that form in a semi-regular pattern in the filament (Figure 2). Vegetative cells of *A. variabilis* can also differentiate into akinetes, which are spore-like cells that survive environmental stress [30]. Nitrogen stress may also induce the formation of motile filaments called hormogonia in *A. variabilis* [19]. In other cyanobacteria hormogonia are required for the establishment of symbiotic associations with plants [31]. *A. variabilis* has oxygen-evolving photosynthesis; however, it is also capable of photoheterotrophic growth and chemoheterotrophic growth in the dark using fructose [23,32-34]. The strain cannot ferment; hence, it does not grow anaerobically in the dark with fructose. The genome sequence revealed the ABC-type fructose transport genes that were subsequently shown to be required for heterotrophic growth of the strain [32].

**Table 1:** Classification and general features *of A. variabilis* ATCC 9413 according to the MIGS recommendations [14]

MIGS ID	Property	Term	Evidence Code
	Current Classification	Domain *Bacteria*	TAS [15]
		Phylum *Cyanobacteria*	
		Class *Nostocophycidae*	
		Order *Nostocales*	
		Family *Nostocaceae*	
		Genus *Anabaena*	TAS [16]
		Species *Anabaena variabilis*	
	Gram stain	Negative	TAS [17]
	Cell shape	Ovoid cells in filaments	TAS [18]
	Motility	Hormogonia	TAS [19]
	Sporulation	Akinetes	TAS [20]
	Temperature range	20 – 40^°^C	TAS [21]
	Optimum temperature	35^°^C	TAS [22]
MIGS-22	Relationship to Oxygen	Aerobic	NAS
	Carbon source	Autotroph, heterotroph	TAS [23]
	Energy source	Phototroph, heterotroph	TAS [23]
MIGS-6	Habitat (EnvO)	Fresh water	TAS [2]
MIGS-6.3	Salinity	1.5% (maximum)	TAS [24]
MIGS-10	Extrachromosomal elements	4	TAS (this report)
MIGS-11	Estimated Size	7.1 Mb	TAS (this report)
MIGS-14	Known Pathogenicity	None	NAS
MIGS-15	Biotic Relationship	Free living; symbiotic	IED
MIGS-4	Geographic Location	Isolated Mississippi, 1964	TAS [2]
MIGS-4.1	Latitude	Not reported	
MIGS-4.2	Longitude	Not reported	
MIGS-4.3	Depth	Not reported	
MIGS-4.4	Altitude	Not reported	

Evidence codes - IDA: Inferred from Direct Assay (first time in publication); TAS: Traceable Author Statement (i.e., a direct report exists in the literature); NAS: Non-traceable Author Statement (i.e., not directly observed for the living, isolated sample but based on a generally accepted property for the species, or anecdotal evidence). These evidence codes are from the Gene Ontology project [25]. If the evidence code is IDA, then the property was directly observed for a living isolate by one of the authors or an expert mentioned in the acknowledgements.

*A. variabilis* is a well-established model organism for heterocyst formation [35,36], nitrogen fixation [21,37,38], hydrogen production [39,40], photosynthesis [41-43], and heterotrophic cyanobacterial growth [9,32,44]. It is unique among the well-characterized cyanobacteria in that it has three sets of genes that encode distinct nitrogenases [19,37,38,45-48]. One is the conventional, heterocyst-specific Mo-nitrogenase, the second is another Mo-nitrogenase that functions only under anoxic conditions in vegetative cells and heterocysts, while the third is a V-nitrogenase that is also heterocyst specific. These nitrogenases are expressed under distinct physiological conditions so that only one nitrogenase is generally functional [19]. The genome sequence has revealed a large 41-kb island of genes that all appear to be involved in synthesis and regulation of the V-nitrogenase, including the genes for the first vanadate transport system to be characterized in any bacterium [49]. The V-nitrogenase of *A. variabilis* has been exploited for its ability to make large amounts of hydrogen as a potential source of alternative energy production [39,40].

### Chemotaxonomy

The Gram-negative cyanobacterial cell wall has not been well characterized; however, it typically contains lipopolysaccharide. In *A. variabilis* the O antigen contains L-acofriose, L-rhamnose, D-mannose, D-glucose, and D-galactose [50]. The cell envelope of the heterocyst differs from vegetative cells in that it also contains an inner laminated glycolipid layer and an outer fibrous, homogeneous polysaccharide layer. In *A. variabilis* the polysaccharide layer comprises a 1,3-linked backbone of glucosyl and mannosyl residues with terminal xylosyl and galactosyl residues. The side branches comprise glucosyl residues having a terminal arabinosyl residue. The inner heterocyst cell wall of almost all strains of *Anabaena* and *Nostoc* consists of a glycolipid comprising 1-(O-hexose)-3,25-hexacosanediol and 1-(O-hexose)-3-keto-25-hexacosanol [51,52]. The lipids of most cyanobacteria comprise monogalactosyldiacylglycerols, digalactosyldiacylglycerols, sulphoquinovosyldiacylglycerols and phosphatidylglycerols [53].

In *A. variabilis* the primary products of lipid biosynthesis are 1-stearoyl-2-palmitoyl species of monoglucosyl diacylglycerol, phosphatidylglycerol and sulfoquinovosyl diacylglycerol; however, the degree of saturation of the fatty acids in the lipids depends on the growth temperature [54-56]

### Genome project history

This organism was selected for sequencing because of its 50-year long history as a model organism for studies on many aspects cyanobacterial metabolism including photosynthesis, nitrogen fixation, hydrogen production, and heterotrophic growth. The genome project is deposited in the Genome On Line Database (Gc00299) and the complete genome sequence is deposited in GenBank. Sequencing, finishing and annotation were performed by the DOE Joint Genome Institute (JGI). A summary of the project information is shown in Table 2.

**Table 2:** Project Information

**MIGS ID**	**Property**	**Term**
MIGS-31	Finishing quality	Finished - < one error per 50 kb
MIGS-28	Libraries used	3-kb pUC18c; 9-kbpMCL200; 40-kb pCC1Fos
MIGS-29	Sequencing platforms	Sanger; ABI3730
MIGS-31.2	Fold coverage	11×
MIGS-30	Assemblers	Phred/Phrap/Consed
MIGS-32	Gene calling method	Critica, Glimmer
	Genbank ID	240292
	Genbank Date of Release	September 17, 2005
	GOLD ID	Gc00299
	Project relevance	Hydrogen production; nitrogen fixation

### Strain history

The strain was first isolated by R.G. Tischer, in 1964 in Mississippi, who called it *Anabaena flos-aquae* MSU A-3 7 [1]. It was submitted to the Indiana University Culture Collection (*Anabaena flos-aquae* IUCC 1444) and was then submitted by C.P Wolk as *Anabaena variabilis* to ATCC in 1976 (*Anabaena variabilis* ATCC 29413). The phylogenetic tree (Figure 1) reveals that the strain clusters with cyanobacteria in the genus *Nostoc*, which is consistent with the fact that it produces hormogonia [19], and not with the cluster of *Anabaena*/*Aphanizomenon*, suggests that the strain was incorrectly named.

### Growth conditions and DNA isolation

An axenic culture of *A. variabilis* ATCC 29413 was grown photoautotrophically in one L of an eight-fold dilution of the medium of Allen and Arnon (AA/8) [57], supplemented 5.0 mM NaNO_3_ at 30°C with illumination at 50-80 μEinsteins m^-2^ s^-1^ to an OD_720_ of about 0.3. Cells were harvested by centrifugation, frozen and then lysed by a combination of crushing the frozen pellet with a very cold mortar and pestle, and then treating the frozen powder with lysozyme (3.0 mg/ml)/proteinase K (1 mg/ml) in 10 mM Tris, 100 mM EDTA pH 8.0 buffer at 37°C for 30 min. This was followed by purification of the DNA using a Qiagen genomic DNA kit. The DNA was precipitated with isopropanol, spooled, and then dissolved in 10 mM Tris, 1.0 mM EDTA pH 8.0 buffer. The purity, quality and size of the bulk gDNA preparation were assessed by JGI according to DOE-JGI guidelines.

## Genome sequencing and annotation

### Sequencing and assembly

Sanger sequencing was done using a whole-genome shotgun approach with three plasmid libraries. A pUC18c library with 3-kb inserts generated 39.64 Mb of sequence. A pMCL200 library with 9-kb inserts produced 35.16 Mb of sequence, and a fosmid(pCC1Fos CopyControl fosmid library production kit; Epicentre, Madison, WI) library with 40-kb inserts yielded 5.83 Mb of sequence. Together, all libraries provided greater than 11.0× coverage of the genome. The plasmid inserts were made with sheared DNA that was blunt-end repaired and then size separated by gel electrophoresis. Sequencing from both ends of the plasmid inserts was done using dye terminators on ABI3730 sequencers. Details on the cloning and sequencing procedures are available from JGI [58]. Project information is summarized in Table 2.

The Phred, Phrap, and Consed software package was used for sequence assembly and quality assessment [59] Repeat sequences were resolved with Dupfinisher [60]. Gaps between contigs were closed by editing in Consed, custom priming, or PCR amplification. This genome was curated to close all gaps with greater than 98% coverage of at least two independent clones. Each base pair has a minimum q (quality) value of 30 and the total error rate is less than one per 50,000.

### Genome annotation

Genes were identified using two gene modeling programs, Glimmer [61] and Critica [62] as part of the Oak Ridge National Laboratory genome annotation pipeline [63].The two sets of gene calls were combined using Critica as the preferred start call for genes with the same stop codon. Genes with less than 80 amino acids that were predicted by only one of the gene callers and had no Blast hit in the KEGG database at 1e^-5^ were deleted. This was followed by a round of manual curation to eliminate obvious overlaps. The predicted CDSs were translated and used to search the National Center for Biotechnology Information (NCBI) nonredundant database, UniProt, TIGRFam, Pfam, PRIAM, KEGG, COG, and InterPro databases. These data sources were combined to assert a product description for each predicted protein. Non-coding genes and miscellaneous features were predicted using tRNAscan-SE [64], TMHMM [65], and signal [66].

## Genome properties

**Figure 3:**
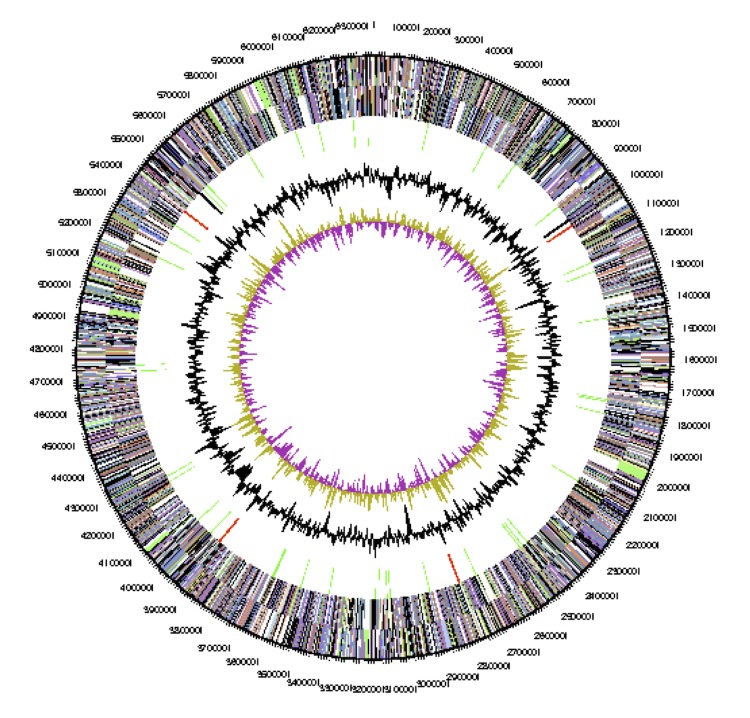
Graphical circular map of the chromosome of *Anabaena variabilis*. From outside to the center: Genes on forward strand (color by COG categories), Genes on reverse strand (color by COG categories), RNA genes (tRNAs green, rRNAs red, other RNAs black), GC content, GC skew.

The genome of *A. variabilis*, with a total of 7.1 Mbp (7,105,752 bp), has 41.4% GC (Table 4). There is a single large circular chromosome (6.36 Mbp) (Figure 3), three circular plasmids and one linear DNA element (Table 3). Plasmid A is circular with 366,354 bp and 41% GC. Plasmid B is circular with 5,762 bp and 38% GC. Plasmid C is circular with 300,758 bp and 42% GC.

**Table 4:** Nucleotide content and gene count levels of the genome

**Attribute**	Value	% of total
Genome size (bp)	7,105,752	100
DNA coding region (bp)	5,849,926	82.3
DNA G+C content (bp)	2,942,474	41.4
Total genes	5,772	100
RNA genes	62	1.0
Protein-coding genes	5,710	99.0
Protein coding genes with function prediction	3,079	53.3
Genes in paralog clusters	3,431	59.4
Genes assigned to COGs	3,670	63.6
Genes assigned Pfam domains	4,352	75.4
Genes with signal peptides	873	15.1
Genes with transmembrane helices	1,383	24.0
CRISPR repeats	7	0.1
Paralogous groups	851	14.6

**Table 3:** Summary of genome: one chromosome, three plasmids and one linear element

Label	Size (Mb)	Topology	INSDC identifier
Chromosome	6.36	circular	NC_007413
Plasmid A	0.366	circular	NC_007410
Plasmid B	0.036	circular	NC_007411
Plasmid C	0.30	circular	NC_007412
Incision element	0.37	linear	NC_014000

**Figure 4:**
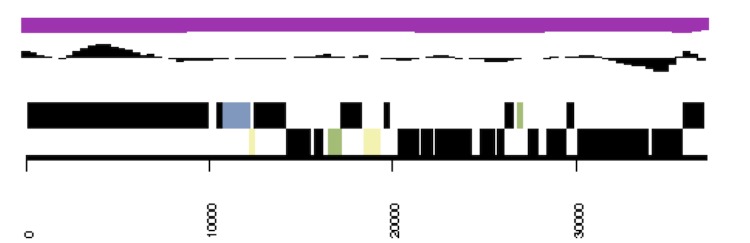
Graphical map of the linear incision element of *Anabaena variabilis*. From the bottom to the top: Genes on forward strand (color by COG categories), Genes on reverse strand (color by COG categories), GC content, GC skew.

The linear incision element is 37,151 bp long with a higher GC content (46%) than the rest of the genome (Figure 4). The incision element has 40 ORFs of which only 5 have any similarity to known genes. AvaD004 has about 40% aa similarity to many proteins provisionally identified as phage terminases, which are involved in phage assembly. AvaD0022 is similar to RNA polymerase sigma factors, with 35% identity to a *sigF* encoded sigma factor, present in many other cyanobacteria including two copies of a similar gene of the large chromosome of *A. variabilis*. AvaD0026, identified as similar to site specific XerD-like recombinases shows 50-55% identity to genes present in many cyanobacteria including the B plasmid of *A. variabilis* and the alpha plasmid of *Anabaena sp.* PCC 7120. AvaD0037 shows similarity to the XRE family of transcriptional regulator and 65% identity to similar proteins in three sequenced strains of the cyanobacterium *Cyanothece*. AvaD0015 is a histone-like DNA binding protein with about 70% identity to the HU gene present in most cyanobacteria including the gene on the large circular chromosome of *A. variabilis*. Many linear molecules overcome the problem of replicating the genome ends using terminal hairpins; however, there is no evidence of such repeats in this element.

A total of 5,772 genes were predicted in the whole genome. Of these, 3,079 were annotated as coding for known protein functions and 62 for RNA genes (12 for rRNA and 50 for tRNA). The distribution of genes into COGs is presented in Table 4. There is considerable redundancy, with 5,710 protein coding genes belonging to 841 paralogous families in this genome.

## Identification of *vnf* genes in other cyanobacterial genomes

**Figure 5:**
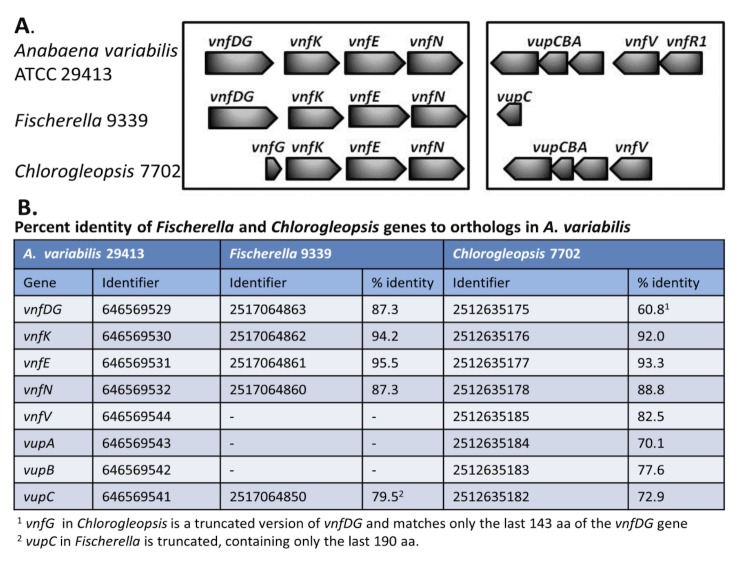
Comparison of the organization of the *vnf* genes for the V-nitrogenase and the *vup* genes for vanadate transport from *A. variabilis* with similar genes found in the genomes of *Fischerella* 9339 (taxon ID 2516653082) and *Chlorogleopsis* 7702 (taxon ID 2512564012), based on the genome sequences available at from the JGI data base. Gene identifiers are the Gene Object ID numbers in the IMG database. **A**. Graphical representation of similar genes. **B**. Percent identity of genes.

The V-nitrogenase is not widespread among bacteria and has, to date, been characterized in only one cyanobacterium, *A. variabilis* [42,49,67], [Figure 5]. Using the large number of cyanobacterial genomes now available, we searched the IMG database for orthologs for the V-nitrogenase genes (*vnf*) and the vanadate transport genes (*vupABC*) present in *A. variabilis*. Only two strains showed any evidence of *vnf* genes, *Fischerella* 9339 (taxon ID 2516653082) and *Chlorogleopsis* 7702 (taxon ID 2512564012). *Fischerella* 9339 has orthologs of *vnfDG*, *vnfK*, *vnfE* and *vnfN*, but is missing most of the vanadate transport genes. In contrast, *Chlorogleopsis* 7702 has orthologs for all three of the vanadate transport genes, and has most of the structural genes for the V-nitrogenase; however, the fused *vnfDG* gene is missing the *vnfD* portion that encodes the alpha subunit of the enzyme, which is essential for dinitrogenase activity. It will be interesting to determine whether either of these strains is capable of fixing nitrogen in the absence of Mo and in the presence of V using the V-nitrogenase.

## Conclusions

*A. variabilis* was one of the earliest model organisms for the study of important cellular processes such as photosynthesis and nitrogen fixation. It is unusual among cyanobacteria in that it has three nitrogenases [19], one of which, the V-nitrogenase, has been shown to be useful for hydrogen production [40], and for its ability to grow both photoautotrophically in the light and heterotrophically in the dark. The genome sequence was critical in identifying the genes for fructose transport [32] and the large island of genes important for V-nitrogenase function, including the *vupABC* genes for vanadate transport [49]. No other cyanobacterial genome has all the genes identified in *A. variabilis* that are important for growth using the V-nitrogenase, but two strains, *Fischerella* 9339 and *Chlorogleopsis* 7702, have some V-nitrogenase or vanadate transport genes. The presence of the linear genetic element shown in Fig. 4 is quite interesting, as such elements are not present in the genomes of the other *Nostoc* strains. It will also be interesting to determine whether this element is important to the cell and how this element replicates.
